# Store-and-forward teledermatology in a Spanish health area significantly increases access to dermatology expertise

**DOI:** 10.1186/s12875-024-02479-1

**Published:** 2024-06-24

**Authors:** Elena Sánchez-Martín, Isabel Moreno-Sánchez, Marta Morán-Sánchez, Miguel Pérez-Martín, Manuel Martín-Morales, Luis García-Ortiz

**Affiliations:** 1 Centro de salud La Alamedilla, Unidad de Investigación en Atención Primaria de Salamanca (APISAL), Gerencia Regional de salud de Castilla y León (SACyL), Avenida de Comuneros 27-31, Salamanca, 37003 Spain; 2 Instituto de investigación Biomédica de Salamanca (IBSAL), Paseo de San Vicente, 58-182, Salamanca, 37007 Spain; 3 Servicio de Pediatría, Hospital La Paz, P.º de la Castellana, 261, Madrid, 28046 España; 4 Departamento de Ciencias Biomédicas y del Diagnóstico, Universidad de Salamanca, Calle Alfonso X el Sabio s/n, Salamanca, 37007 Spain; 5 Red de Investigación en Cronicidad, Atención Primaria y Promoción de la Salud (RICAPPS), Avenida de Portugal 83, Salamanca, 37005 Spain

**Keywords:** Telemedicine, Teledermatology, Skin diseases, Primary care, Dermatology

## Abstract

**Introduction:**

Teledermatology is the practice of dermatology through communication technologies. The aim of this study is to analyze its implementation in a Spanish health area during its first two years.

**Methods:**

Cross-sectional descriptive study. It included interconsultations between dermatologists and family physicians in the Salamanca Health Area (Spain) after the implementation of the non-face-to-face modality over a period of two consecutive years. A total of 25,424 consultations were performed (20,912 face-to-face and 4,512 non-face-to-face); 1000 were selected by random sampling, half of each modality. Main measures: referral rate, response time and resolution time, type of pathology, diagnostic concordance, and quality of consultation.

**Results:**

The annual referral rate was 42.9/1000 inhabitants (35.3 face-to-face and 7.6 non-face- to-face). The rate of face-to-face referrals was higher in urban areas (37.1) and the rate of non- face-to-face referrals in rural areas (10.4). The response time for non-face-to-face consultations was 2.4 ± 12.7 days and 56 ± 34.8 days for face-to-face consultations (*p* < 0.001). The resolution rate for non-face-to-face consultations was 44%. Diagnostic concordance, assessed by the kappa index, was 0.527 for face-to-face consultations and 0.564 for non-face-to-face consultations. Greater compliance with the quality criteria in the non-attendance consultations.

**Conclusions:**

Teledermatology appears to be an efficient tool in the resolution of dermatological problems, with a rapid, effective, and higher quality response for attention to skin pathologies.

**Registry:**

ClinicalTrials.gov Identifier: NCT05625295. Registered on 21 November 2022 (https://clinicaltrials.gov/ct2/show/ NCT05625295).

**Supplementary Information:**

The online version contains supplementary material available at 10.1186/s12875-024-02479-1.

## Introduction

Teledermatology (TD) is a telemedicine technique which consists of skin lesion assessment by dermatologists, allowing the diagnosis and treatment of patients remotely. There are different types: (1) Real-time or synchronous TD, usually via videoconference, where at least two individuals communicate synchronously, i.e., in real-time interaction. The concept can be applied equally well to both a videoconferencing system and simple telephone calls. (2) Delayed TD, store-and-forward or asynchronous TD, where the information and the photographic image is stored and the dermatologist answers in a second communication by e-mail or web access. The previously collected information (patient history and photographic images) is stored and sent to the dermatologist, who responds at a later point. There is no real time interaction between patient and dermatologist. Email or web access can be used, which allows a medical history to be attached, with the minimum essential items, together with the digital images of the patient’s skin lesions. The procedure is asynchronous. There are also hybrid formats [1]. Currently, the most widely used is store-and-forward TD as it is more efficient and easier to coordinate [2].

Studies on the validity of TD [3, 4] have found it to be a reliable, effective and efficient consultation instrument, and a practical tool for the family doctor [5, 6]. TD improves patient accessibility to the dermatologist, avoids long distances and transmits information quickly [7]. It also makes it possible to prioritize the diagnosis of skin cancer [8] and any other skin pathology [9], and contributes to better coordination between care levels, reducing costs and waiting lists [10, 11].

TD also has some disadvantages, such as: loss of relationship with the patient, limitation of clinical information to forms, need to retake and resubmit poor quality images, thus generating further delays and resistance to change among professionals.

TD is more established in better developed countries in Europe, America and Southeast Asia [3]. In Spain, there are large differences between autonomous communities [12, 13], with greater implementation in Catalonia, the Basque Country, Madrid, Galicia, Castilla la Mancha and Andalucía, where storage TD predominates [14]. The Covid-19 pandemic provided a boost and has accelerated its use and utility both in Spain and in other parts of the world [15, 16].

Evaluation of the effectiveness and efficiency of this technology is essential to guarantee the objectives of its implementation [17, 18]. In Spain, several studies have been carried out to evaluate the use of TD in clinical practice [19–21] with satisfactory results, but none in the Castilla y León region.

The Spanish national health system is managed by public entities, with 90% of the population enrolled. It has three organizational levels: national, autonomous and local health area. The latter has a reference hospital for specialized care. The health area is divided into basic health zones, with a health center (physical structure for globally coordinated, comprehensive, and permanent primary care) where primary care professionals work (family doctors, nurses, physiotherapists, etc.). Primary care is the basic and initial level of patient care, ensuring comprehensive and continuous life-long care. Healthcare is provided on demand, scheduled or urgent, either in the center or at the patient’s home by prior appointment. In the Spanish national health system, each person is assigned a family doctor in a health center which can be easily accessed by appointment. This doctor ensures globally coordinated, comprehensive, and permanent primary care, making an initial assessment of the health problem and, if necessary, referring patients to specialists such as dermatologists.

The aim of this study is to evaluate the implementation of TD, in the form of non-face-to-face consultation, in a health area of the autonomous community of Castilla y Leon (Spain) and to describe its use by family physicians and dermatologists, as well as its effectiveness in improving response time and problem resolution, and the quality of care provided by the family physician and dermatologist.

## Methods

### Design and setting

Cross-sectional descriptive study of the interconsultations between family doctors and dermatologists in the Salamanca health area (Castilla y Leon, Spain) to analyze the implementation of TD consultation in the first two years of operation. This health area provides health services to 328,417 inhabitants, in 36 health centers and a tertiary hospital with a dermatology service. During the study period, there were a total of 12 dermatologists for the entire health area, a ratio of 1 dermatologist for every 24,613 persons. The waiting time for a first face-to-face consultation was 56 +/-34.6 days, reduced to 7–15 days in preferential consultations.

### Patients

**Fig. 1:**
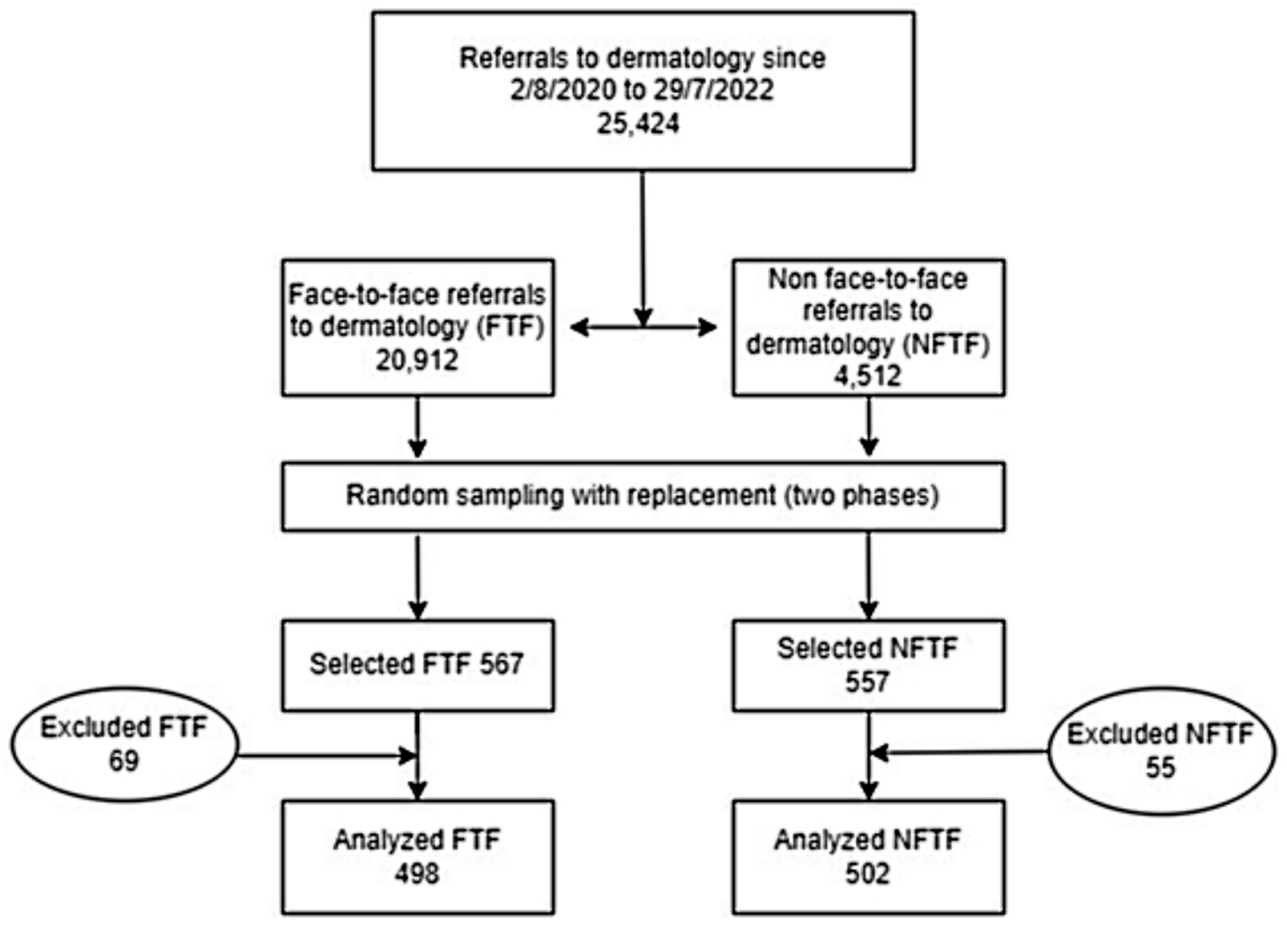
Flow chart diagram of the selection of patients included in the study. Reasons for missing cases in both groups were: lack of information in the clinical history that would allow assessment of the consultation, no family doctor assigned in the Salamanca area, and death of the patient, which did not allow access to the clinical history. FTF: face-to-face consultation. NFTF: non-face-to-face consultation

We included all dermatological consultations requested by patients aged 14 years or older by family doctors between August 2, 2020 and July 29, 2022. A total of 25,424 interconsultation requests (IC) were recorded during this period, of which 20,912 (83.3%) were face-to-face (FTF-IC) and 4.512 (17.7%) were non-face-to-face (NFTF-IC). The sample size was estimated to detect as significant a difference of 7 days in the response time between face-to-face and non-face-to-face consultation. Accepting an alpha risk of 0.05 and a beta risk of 0.2 in a bilateral contrast, 488 subjects were required in each group, assuming a common standard deviation of 37 days and estimating a loss rate of 10%. By random sampling with replacement, 1000 subjects with dermatology consultation were selected (498 FTF-IC and 502 NFTF-IC). We excluded 124 subjects (55 NFTF-IC and 69 FTF-IC) who met one of the following criteria: no information in their medical records, no physician assigned in Salamanca or death during the period evaluated (Fig. 1).

### Non-face-to-face interconsultation procedure

This study analyzes asynchronous TD consultations. The tele-consultation (clinical data and images) is created, sent, evaluated and answered completely independently in time. The family physician sends the patient’s data and the dermatological lesion or pathology to be consulted to the dermatologist from the computerized primary care medical record, together with the contribution of photographic images to be assessed by the specialist. The dermatologist responds at a later point with his/her diagnostic judgment and resolution of the process if necessary, or makes an appointment with the patient for a face-to-face consultation.

It is the family physician who is responsible for deciding on and prioritizing the request for a non-face-to-face consultation, mainly for lesions suspected to be skin cancer and those that can be easily photographed for the image to be forwarded to the dermatologist.

### Measurement

The information regarding the variables described below was collected from the computerized medical records of primary care (Medora) and specialized care (Jimena) by six researchers, previously trained to homogenize the collection criteria. The patients’ health centers were classified by population: urban (over 100,000 inhabitants), semi-urban (between 5,000 and 100,000) and rural (under 5,000), and by age and sex of the patients.

Dermatology consultation: type of consultation, date of request, date of first response and resolution, need for dermatology follow-up, use of dermatoscopy, diagnosis coded according to ICD 10 for the family doctor and according to DIADERM study [22] criteria for the dermatologist (Table S1: Supplementary material). The quality of the photography was evaluated according to the recommendations of the American Teledermatology Association (ATA) [23] (Table S2: Supplementary material).

The evaluation of the interconsultation quality used the following criteria: In the family doctor´s request: (1) Indication of reason for consultation; (2) Provision of images; (3) Description of the skin process: shape, borders, size, color, location, onset, accompanying symptoms and previous treatments. In the dermatologist’s response: (1) Diagnostic approach; (2) Therapeutic, medical, or surgical orientation; and (3) Appointment for a face-to-face dermatology consultation.

### Statistical analysis

Data were recorded using the REDCap (Research Electronic Data Capture) platform [24], with a questionnaire previously designed for the project. The normal distribution of quantitative variables was verified using the Kolmogorov-Smirnov test. The analysis of the difference in means between qualitative variables in two categories was carried out by Student’s t-test for independent groups and for more than two categories by analysis of variance. For the contrast of qualitative variables, the X^2^-test was used. Concordance in diagnosis between family doctor and dermatologist was analyzed using the kappa index. Data were analyzed using SPSS for Windows, version 25.0. A value of *p* < 0.05 was considered statistically significant.

## Results

The mean age of dermatology consultation patients was 54.5 ± 22.2 years, more specifically 53.9 ± 22.1 years in face-to-face and 56.8 ± 22.5 years in non-face-too-face consultations (*p* < 0.001); 58.5% were women, with no difference in the type of consultation. Table 1 shows the total number of referrals for the evaluation period, as well as the annual referral rate per 1000 population overall and according to health center characteristics, age and sex. The rate of face-to-face consultation was higher in urban (37.1) and semi-urban (36.7) health centers than in rural (27.2) ones, and the non-face-to-face rate was higher in rural (10.4), compared to 6.5 and 8.2 in urban and semi-urban, respectively. There was no gender difference in the type of consultation, but an age difference was found, with face-to-face patients being 2.78 (95% CI: 2.06 to 3.48) years younger.

**Table 1:** Dermatological interconsultations by health center, age and sex

						No.				%		Annual rate per 1000 pop	
Type of health center		Urban (pop > 100,000)				13,000				51.1		43.6	
		Semi-urban (pop 5,000-100,000)				9,607				37.8		43.9	
		Rural (pop < 5,000)				2,817				11.1		37.6	
		Total				25,424				100.0		42.9	
FTF-IC		Urban (pop > 100,000)				11,059				52.9		37.1	
		Semi-urban (pop 5,000-100,000)				7,814				37.4		35.7	
		Rural (pop < 5,000)				2,039				9.8		27.2	
		Total				20,912				100.0		35.3	
NFTF-IC		Urban (pop > 100.000)				1,941				43.0		6.5	
		Semi-urban (pop 5,000-100,000)				1,793				39.7		8.2	
		Rural (pop < 5,000)				778				17.2		10.4	
		Total				4,512				100.0		7.6	
								**Mean/n**		**SD/%**		**p**	
Age				FTF-IC		54.0			22.1			< 0.001	
				NFTF-IC		56.8			22.5				
				Total		54.5			22.2				
Sex		Male		FTF-IC		8,679			82.2			0.907	
				NFTF-IC		1,877			21.6				
				Total		10,556			100.0				
		Female		FTF-IC		12,233			82.3				
				NFTF-IC		2,635			21.5				
				Total		14,868			100.0				

FTF-IC: face-to-face interconsultation. NFTF-IC: non-face-to-face interconsultation. Quantitative variables are expressed with mean and standard deviation (SD), qualitative variables are expressed with absolute frequency and percentage

Table 2 shows the socio-demographic characteristics of the 1,000 patients selected. In face-to-face cases, 50.6% were from urban and 9.4% from rural areas, while in non-face-to-face, 44.3% of patients were from urban and 19.1% from rural areas. The mean age of the sample was 55.5 ± 21.9 years, and 57.7% were women. In the selected sample, no differences were found in the type of consultation, age or sex, but there was a difference in the type of health center. In rural centers, 67.1% were non-face-to-face, while in urban and semi-urban centers the figure was less than 50% (*p* < 0.001).

**Table 2:** Characteristics of sample selection

Type of health center							*n*		%		
		Urban (pop > 100,000)					475		47,5		
		Semi-urban (pop 5,000-100,000)					382		38,2		
		Rural (pop < 5,000)					143		14,3		
		Total					1,000		100.0		
**Type of interconsultation**							n		%		
		FTF-IC					498		49.9		
		NFTF-IC					502		50.1		
FTF-IC		1. Urban (pop > 100,000)					252		50.6		
		2. Semi-urban (pop 5,000-100,000)					199		40.0		
		3. Rural (pop < 5,000)					47		9.4		
NFTF-IC		1. Urban (pop > 100,000)					223		44.4		
		2. Semi-urban (pop 5,000-100,000)					183		36.5		
		3. Rural (pop < 5,000)					96		19.1		
**Age and sex**							n/mean		%/SD		
Age of patient (mean)							55.5		21.9		
Sex of patient		Male					422		42.2		
		Female					578		57.8		
Sex of family doctor		Male					331		33.1		
		Female					669		66.8		
Sex of dermatologist		Male					299		29.9		
		Female					701		70.0		
**Sociodemographic variables and type of interconsultation**											
			**Type of interconsultation**								
				**FTF-IC**		**NFTF-IC**					
				**Mean/n**	**SD/%**	**Mean/n**		**SD/%**		**p**	
Age				55.4	21.5	55.6		22.2		0.876	
Age group	< 44 years			170	51.2	162		48.8		0.783	
	44–67 years			167	49.9	168		50.1			
	> 67 years			161	48.3	172		51.7			
Sex	Male			210	49.8	212		50.2		0.503	
	Female			288	49.8	290		50.2			
Type of health center	Urban (pop > 100,000)			252	53.1	223		46.9		< 0.001	
	Semi-urban (pop 5,000-100,000)			199	52.1	183		47.9			
	Rural (pop < 5,000)			47	32.9	96		67.1			

FTF-IC: face-to-face interconsultation. NFTF-IC: non-face-to-face interconsultation. Quantitative variables are expressed with mean and standard deviation (SD), qualitative variables are expressed with absolute frequency and percentage

Response time, from the request for consultation by the family doctor to the first response or visit by the dermatologist, was 29.1 ± 37.5 days on average. The response time for face-to-face was 56 ± 34.8 days and 2.4 ± 12.7 days for non-face-to-face (*p* < 0.01). Overall days to resolution was 83 ± 120.7, with 108.2 ± 119.7 days in face-to-face and 58.2 ± 116.7 days in non-face-to-face (*p* < 0.001). Resolution in face-to-face, assessed as solved on the day the dermatologist answered for the first time, was 44.6% of the total, with the rest requiring referral to the dermatologist.

**Fig. 2:**
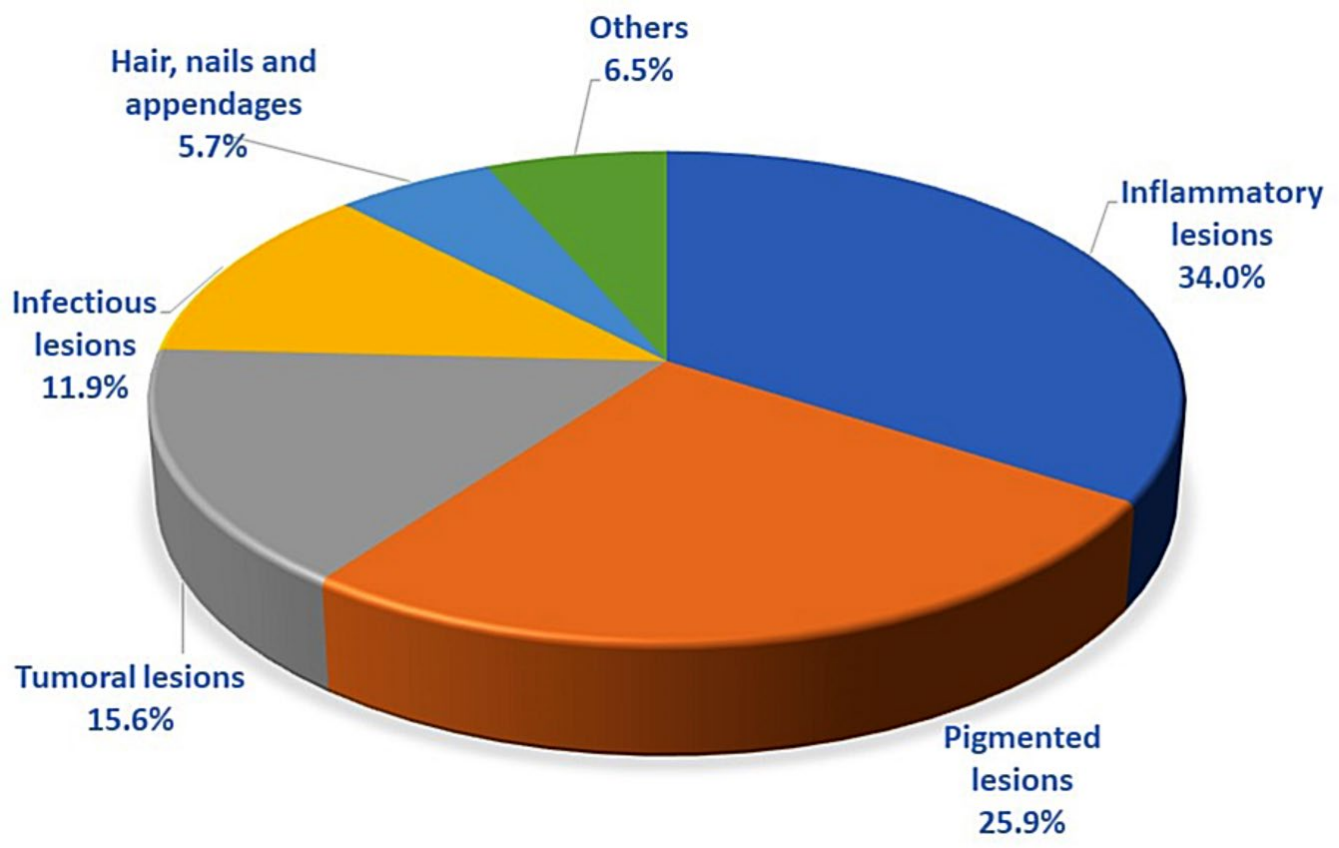
Type of lesions according to the family doctor’s clinical suspicion

Figure 2 shows the type of pathology referred according to the family doctor’s diagnostic suspicion in order of frequency. The main types of lesions were: inflammatory 34%, pigmented 25.9%, tumoral 15.6% and infectious 11.9%, with similar rates in both types of consultation.

Dermoscopy was used for family practice in 2.6% of all consultations, with greater use in non-face-to-face (4.8%) than in face-to-face cases (0.4%). The use of dermoscopy by the dermatologist was not evaluated. Photographic images were sent in 93.4% of non-face-to-face cases, with poor assessment in 23.8%, acceptable in 46.4% and good in 29.8% of cases, according to ATA criteria.

Table 3 shows the diagnoses of the general practitioner and dermatologist according to ICD 10 categories. The most frequent diagnostic suspicion for the general practitioner was pigmented lesions (9.9%), viral warts (5.3%) and seborrheic keratosis (5.6%), while for the dermatologist it was seborrheic keratosis (12.2%), pigmented lesions (7.7%) and non-melanoma skin cancer (7.4%). The diagnostic concordance analyzed by the kappa index was 0.546 (moderate grade), with 0.527 in face-to-face and 0.564 in non-face-to-face cases.

**Table 3:** ICD 10 codes for family doctor and dermatologist diagnoses

		*n*	%
Family doctor	1 L57 - Skin disorders	51	5.1
	2 C44 - Non-melanoma skin cancer	47	4.7
	3 D22 - Melanocytic nevi	99	9.9
	4 L82 - Seborrheic keratosis	56	5.6
	5 D23 - Other benign skin neoplasms	22	2.2
	6 L40 - Psoriasis	21	2.1
	7 L70 - Acne	47	4.7
	8 B07 - Viral warts	52	5.3
	9 L81 - Other pigmentation disorders	45	4.5
	10 L30 - Other and unspecified dermatitis	72	7.2
	11 L20 - Atopic dermatitis	6	0.6
	12 L50 - Urticaria	10	1.0
	13 Other	459	45.9
	Subtotal	988	98.7
	Missing	13	1.3
	Total	1,000	100
Dermatologist	1 L57 - Skin disorders	65	6.5
	2 C44 - Non-melanoma skin cancer	74	7.4
	3 D22 - Melanocytic nevi	77	7.7
	4 L82 - Seborrheic keratosis	122	12.2
	5 D23 - Other benign skin neoplasms	18	1.8
	6 L40 - Psoriasis	19	1.9
	7 L70 - Acne	45	4.5
	8 B07 - Viral warts	32	3.2
	9 L81 - Other pigmentation disorders	33	3.3
	10 L30 - Other and unspecified dermatitis	63	6.3
	11 L20 - Atopic dermatitis	3	0.3
	12 L50 - Urticaria	8	0.8
	13 Other	402	40.2
	Subtotal	962	96.1
	Missing	38	3.9
	Total	1,000	100

**Fig. 3:**
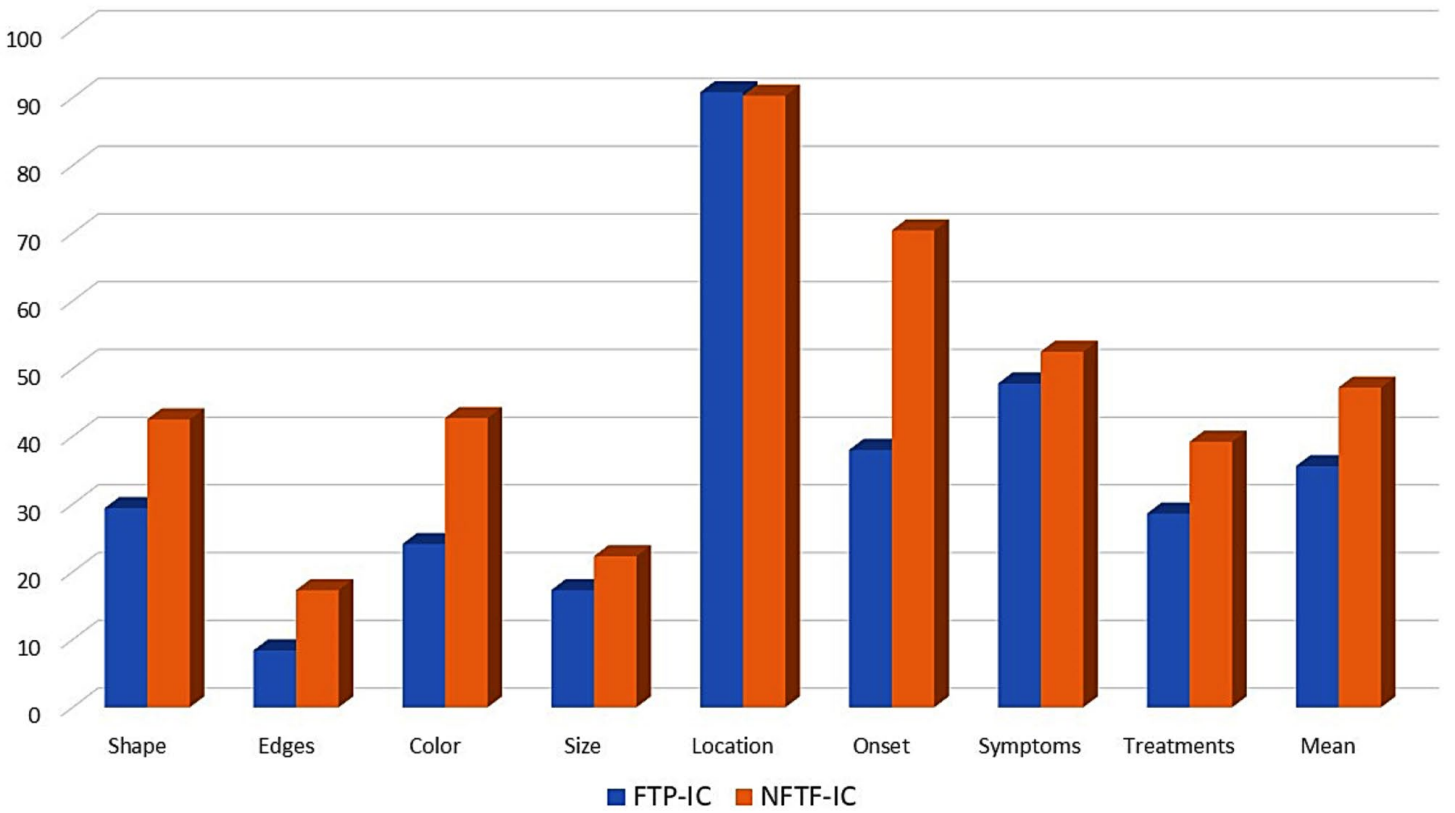
Quality criteria of the family doctor’s interconsultation: description of the skin process. *Note P* < 0.05 for mean score and all criteria except location and accompanying symptoms. NFTF-IC: non-face-to- face inter-interconsultation. FTF-IC: face-to-face interconsultation

In the quality assessment of the family doctor’s consultation, the reason for consultation was specified in 97.1% of cases (96.8% of face-to-face and 97.6% of non-face-to-face), and images were provided in 94% of non-face-to-face and 11.1% of face-to-face consultations. Regarding the description of the cutaneous process, a higher percentage of the criteria were met in non-face-to-face (47.2%) than in face-to-face consultations (35.6%) (*p* < 0.001). The location of the lesion was the most common finding in 90.5% of both types of interconsultation, followed by the onset of the lesion (54.2%) and the existence of accompanying symptoms (50.1%) (Fig. 3).

In the evaluation of the quality of the dermatologist’s response, the diagnostic approach was found in 89.8% of face-to-face (91.8% in non-face-to-face and 87.8% in face-to-face) (*p* < 0.021); treatment guidance was given in 88.2% of consultations (89.5% in non-face-to-face and 86.9% in face-to-face), and dermatology consultations were cited in 74.7% (98.6% in face-to-face and 51.1% in non-face-to-face) (*p* < 0.001).

## Discussion

The annual rate of non-face-to-face utilization per 1,000 population was higher in rural areas than in urban and semi-urban areas. The time to first response and resolution of the problem was shorter in NFTF-IC. Diagnostic concordance between family doctor and dermatologist, although moderate, was slightly higher in non-face-to-face than in face-to-face cases, as was the quality of the consultation of both family doctor and dermatologist. Therefore, non-face-to-face consultation achieved a reduction in time taken to resolve the dermatological problem and improved quality of care.

TD has demonstrated advantages such as reliability and diagnostic accuracy in dermatological pathology [25, 26], reduced time for problem resolution, reduced referrals to dermatology consultation [27] and reduced waiting lists [28]. In our study, the first response time by the dermatologist to non-face-to-face was 2.4 days with an average resolution time of 58.1 days, compared to 55.9 days for patients waiting to be assessed in face-to-face cases, and a resolution time of 108.2 days. The response time in non-face-to-face is similar to that described in an area of Granada, (Spain), stipulated at 2 days and 20 h, according to Ayén-Rodriguez et al. [20], and in a health area in the United States, where the median was 1 day (IQR 1–2) [29]. A further advantage observed was the reduction of costs in dermatological care [28]; although in this study we did not evaluate this aspect, a significant reduction was noted in the number of hospital consultations, loss of working days in the working population, and transport costs for patients who did not live in the city where the hospital was located. This study clearly supports the prioritization of non-face-to-face consultation, facilitating accessibility through this type of interconsultation for inhabitants of rural areas.

Along similar lines, the study by Batalla et al. [30] shows that TD avoids half of the referrals from primary care, with a greater capacity for resolution in the group of infectious diseases, inflammatory diseases, tumors and benign pigmented lesions. The EVIDE-19 Study [21], which used an app to send images of skin lesions to the dermatologist, concluded that 17% of patients were able to avoid face-to-face consultations and 68% of patients were able to delay face-to-face consultations for at least 3 months. The rate of resolution of non-face-to-face in our sample was higher, reaching 44.6%, so that just over 50% needed assessment at the hospital. These results are similar to those obtained by Vaño-Galván et al. [12]. , in which 40% of face-to-face consultations were avoided and in that of Pasdyn et al. [29], in which they recommended that 45% of the non-face-to-face visits be conducted face-to-face.

In the DIADERM study [22], the first to analyze the diagnoses of patients seen in dermatology outpatient clinics nationwide, the most frequent diagnoses were actinic keratosis, basal cell carcinoma and melanocytic nevus. These data are very similar to those found in our study, in which 33.2% of the family doctor’s initial diagnostic suspicions were for inflammatory lesions, 25.9% for pigmented lesions and 15.6% for tumor lesions.

Diagnostic concordance between family doctors and dermatologists in this study was moderate, although in line with other studies analyzing this concordance [29, 31], with similar or worse results. Dermatoscopy was only used in 2.6% of IC, predominantly in non-face-to-face, with a consequently low contribution of dermatoscopic images which facilitate diagnosis for the dermatologist [32]. Better training in dermatological pathology and greater use of dermatoscopy in the primary care setting could improve this aspect, since, as has been shown in this study, its use is still scarce in family doctor´s surgeries. This would improve the diagnostic accuracy of dermatological lesions and the early detection of skin cancer [33], as well as the quality of the photographs [34]. Diagnostic concordance between dermatologists in face-to-face and teledermatology consultations has also been analyzed in some studies, such as that by Emily L. Clarke [6], in which the overall concordance in first diagnosis was good (kappa 0.60), which reinforces the validity of this type of interconsultation.

With regard to the quality of the consultation, although in general there is room for improvement in both types of consultation, the quality criteria scores are higher in non-face-to-face than in face-to-face, both in the family physician’s request and in the dermatologist’s response. The description of the lesion is where the greatest problems were found, especially in face-to-face, although neither of the two (47.2% in non-face-to-face and 35.2% in face-to-face) reached half of the possible points. This would indicate that the use of TD, in addition to improving response times, can be a tool for improving the quality of care. Dermatologists also have a positive perception of the usefulness of TD and are in favor of its application [35].

In summary, the assessment of the NFTF by the dermatologist in 2.4 days is an excellent achievement for professionals and patients. This noteworthy advance in organizational capacity improves appointment management, optimizes the care process, adjusts work schedules, allows greater and better patient accessibility, provides greater user satisfaction and a better perception of the health status of the population. TD is presented as a more accessible alternative that can be quickly implemented to improve the speed of patient care, reduce waiting lists and reduce the pressure of care, while also reducing costs. These results obtained in TD show it to be a digital management tool for medical services that streamlines processes and acts as a highly effective and highly valued complement to face-to-face consultations, improving the patient experience and resolving cases effectively and efficiently.

There are several limitations to this study. It has a retrospective design, with the information recorded in the clinical records by many health professionals, so there may be a deficit of records, and this information was not assessed. The evaluators were several health professionals, and although training was carried out to homogenize criteria, there may be some discrepancies in the interpretation of the information. The number of people excluded, although not high (12.4%), may also have influenced the data analyzed.

## Conclusion

The use of non-face-to-face consultation in dermatology speeds the dermatologist’s first response and the resolution of the problem, and reduces the need for face-to-face consultation at the hospital. It also improves diagnostic concordance between family doctor and dermatologist and the quality of care provided by both professionals.

### Electronic supplementary material

Below is the link to the electronic supplementary material.

Supplementary Material 1

Supplementary Material 2

## Data Availability

Data are available upon request.

## Abbreviations

NFTF-IC: non-face-to- face inter-consultation
FTF-IC: face-to-face consultation
TD: teledermatology
