# Supplementary material for: Store-and-forward teledermatology in a Spanish health area significantly increases access to dermatology expertise
Source: BMC Prim Care. 2024 Jun 24;25:227. doi: 10.1186/s12875-024-02479-1 (PMC11197177; doi:10.1186/s12875-024-02479-1)
Supplement: Supplementary file 1 — Supplementary Material 1 [file 12875_2024_2479_MOESM1_ESM.docx]

Supplementary Material

# Table S1: Distribution of the estimated diagnoses in Spain according to ICD-10 categories (DIADERM Study) (1)

L57: Skin disorders (actinic keratosis, etc.)

C44: Non-melanoma skin cancer (basal cell carcinoma, etc.)

D22: Melanocytic nevi

L82: Seborrheic keratoses

D23: Other benign skin neoplasms (nevus)

L40: Psoriasis

L70: Acne

B07: Viral warts (verruca vulgaris)

L81: Other pigmentation disorders (solar lentigo/Melasma)

L30: Other dermatitis and unspecified dermatitis (eczema SAI)

L20: Atopic dermatitis

L50: Urticaria

Others Otros

# Table S2: American Telemedicine Association recommendations for performing tele dermatology photography (2).

**For a generalized rash:**

1. Anterior and posterior body imaging to distribute information 2.
2. Normal examination distance of typical lesions (22.86 to 30.48cm).
3. For very specific lesions, use extreme close-up with the macro objective at no less than 10.16cm away from the surface of the lesion (always identify the area of the body beingexamina)

# For individual lesions:

1. Normal photographic distance (from 22.86 to 30.48cm).
2. Use macro for very specific lesiones approximately 10.16 away from the surface of the lesion (identifyinf the body part being photographed)

**General recommendations**

1. Always use a reference in centimeters present in the photograph, to know the size of the lesion
2. Whenever posible, use a blue or gray curtain as a background
3. Even if there is a Good ambient light, use the flash

**References**

1. Buendía‐Eisman A, Arias‐Santiago S, Molina‐Leyva A, Gilaberte Y, Fernández‐Crehuet P, Husein‐ElAhmed H, et al. Outpatient Dermatological Diagnoses in Spain: Results From the National DIADERM Random Sampling Project. Actas Dermosifiliogr (Engl Ed). 2018;109:416‐23, [http://dx.dpi.org/10.1016/j.ad.2018.02.003.](http://dx.dpi.org/10.1016/j.ad.2018.02.003)
2. Romero G, Garrido JA, García‐Arpa M. [Telemedicine and teledermatology (I): concepts and applications]. Actas Dermosifiliogr. 2008;99:506‐22.
