# Supplementary material for: Store-and-forward teledermatology in a Spanish health area significantly increases access to dermatology expertise
Source: BMC Prim Care. 2024 Jun 24;25:227. doi: 10.1186/s12875-024-02479-1 (PMC11197177; doi:10.1186/s12875-024-02479-1)
Supplement: Supplementary file 2 — Supplementary Material 2 [file 12875_2024_2479_MOESM2_ESM.pdf]

COMPLEJO ASISTENCIAL UNIVERSITARIO D SALAMANCA
Paseo de San Vicente, 58-182
37007 Salamanca
Comité Ético de Investigación con Medicamentos
Teléfono: 923 29 11 00 – Ext. 55 515

Sacyl

E-mail: comite.etico.husa@saludcastillayleon.es

DICTAMEN DEL COMITE DE ETICA DE LA INVESTIGACION CON MEDICAMENTOS DEL AREA DE SALUD DE SALAMANCA

Dña. Mª Belén Vidriales Vicente Secretaria técnica del Comité de Ética de la Investigación con medicamentos del Área de Salud de Salamanca,

C E R T I F I C A

Que este Comité, en su reunión del
ha evaluado el Proyecto de Investigación titulado

Evaluación de la implantación de la tele dermatología, en su modalidad de interconsulta no presencial en la provincia de Salamanca

HIP y CI Version1 de 30 de junio de 2021

Código CEIm: PI **2021 07 852**

del que es Investigador Principal Dña Elena Sánchez Martín

del Servicio de Atención Primaria

valorado de acuerdo con la Ley 14/2007 de Investigación Biomédica, Principios éticos de la Declaración de Helsinki de la Asociación Médica Mundial sobre principios éticos para investigaciones médicas con seres humanos, así como el resto de principios éticos y normativa legal aplicable en función de las características del estudio,

Considera que dicho estudio cumple los requisitos necesarios y es viable para su realización en este centro, por lo que **INFORMA FAVORABLEMENTE** para la realización de dicho estudio

Y para que conste, lo firma en Salamanca con fecha 26 de julio de 2021

MARIA BELEN VIDRIALES VICENTE - DNI 07851455Z

Firmado digitalmente por MARIA BELEN VIDRIALES VICENTE - DNI 07851455Z
Fecha: 2021.07.27 11:47:48 +02'00'

LA SECRETARIA

Fdo.: Dra. Dña. Mª Belén Vidriales Vicente

Composición del CEIm del Área de Salud de Salamanca
Presidente: D. Luis Muñoz Bellvís (Jefe de Servicio de Cirugía General y Aparato Digestivo)
Vicepresidente: D. Enrique Nieto Manibardo (Delegado de protección de datos del CAUSA)
Secretaria: Dña. María Belén Vidriales Vicente (Jefe de Sección de Hematología. Representante de la Comisión de Investigación IBSAL). Vocales: D. Ricardo Tostado Menéndez (Farmacólogo Clínico); Dña. Silvia Jiménez Cabrera (Farmacia Hospitalaria); Dña. Ascensión Hernández Encinas (Profesora Titular Matemática aplicada, Universidad de Salamanca. Presidenta ASCOL, representante de los pacientes); Dña. Mª Teresa Arias Martín (Enfermera de Salud Mental. Miembro del Comité de Bioética Asistencial); Dña. Mª del Carmen Arias de la Fuente (Técnico Gestor de Ensayos Clínicos); Dña. Berta Bote Bonaechea (Especialista en Psiquiatría); Dña. Ángela Rodríguez Rodríguez (Responsable Unidad de Enfermera. S. de Hematología); Dña. Cristina Hidalgo Calleja (Especialista de Reumatología); D. Guzmán Franch Arcas (Especialista en Cirugía General y Aparato Digestivo); D. Antonio Márquez Vera (Fisioterapeuta); Dña. Ana Martín García (Especialista en Cardiología); Dña. Teresa Martín Gómez (Especialista en Oncología); Dña. Concepción Rodríguez Barrueco (Farmacéutica de Atención Primaria); Dña. Carmen Velayos Castelo (Profesora Titular Ética y Filosofía Política, Universidad de Salamanca); D. Manuel Ángel Gómez Marcos (Médico de Atención Primaria. Responsable de la Unidad de Investigación de Atención Primaria de Salamanca)
